# Assessing the Risks for Poliovirus Outbreaks in Polio-Free Countries — Africa, 2012–2013

**Published:** 2013-09-20

**Authors:** World Health Organization, Regional Office for Africa, Global Immunization Div, Center for Global Health, CDC

In 2012, the World Health Assembly of the World Health Organization (WHO) declared the completion of polio eradication a programmatic emergency (1). Indigenous wild poliovirus (WPV) transmission remains uninterrupted in Nigeria (in the WHO African Region [AFR]) and in Afghanistan and Pakistan (in the WHO Eastern Mediterranean Region [EMR]). In the WHO AFR, multiple WPV outbreaks have occurred since 2003 after importation of indigenous West African WPV into 21 previously polio-free countries in a “WPV importation belt”* that extends across the continent (2–3). The Global Polio Eradication Initiative (GPEI) and WHO regional offices have used indicators of population immunity, surveillance quality, and other factors (e.g., high-risk subpopulations and proximity to WPV-affected countries) to assess the risk for outbreaks in polio-free countries and guide the implementation of risk mitigation measures to limit poliovirus transmission after WPV importation and prevent the emergence of circulating vaccine-derived poliovirus (cVDPV) (4). Despite risk mitigation efforts, a polio outbreak, first confirmed in May 2013, is ongoing; as of September 10, a total of 178 WPV type 1 (WPV1) cases have been reported in Somalia† (163 cases), Kenya (14 cases) and Ethiopia (1 case), after importation of WPV1 of West African origin (5). This report summarizes steps taken by the GPEI to assess and mitigate the risks for outbreaks after WPV importation or the emergence of cVDPV in polio-free countries within the WHO AFR’s “WPV importation belt.” All countries will continue to have some level of risk for WPV outbreaks as long as endemic circulation continues in Afghanistan, Nigeria, and Pakistan.

## Risk Assessment

GPEI partners, including WHO regional office teams, have assessed the risks for WPV outbreaks and cVDPV emergence in polio-free countries to support planning and prioritization of risk mitigation activities; a harmonized risk assessment approach has been used across WHO regions since 2011 (2). Risk assessments are based on three broad criteria: 1) population immunity, 2) quality of acute flaccid paralysis (AFP) surveillance, and 3) other population-specific factors, such as the proximity to areas with active WPV transmission, history of previous outbreaks, capacity to respond to outbreaks, presence of nomadic or other high-risk subpopulations or areas, and insecurity or civil unrest. Population immunity is assessed by estimates of vaccination coverage by age 12 months with 3 doses of trivalent oral poliovirus vaccine (tOPV) and the reported number of OPV doses administered among children aged 6–59 months with nonpolio AFP (Figure). The quality of AFP surveillance is assessed by the proportion of provinces/states that achieve an annual nonpolio AFP reporting rate of ≥2 cases per 100,000 population aged <15 years and the proportion of provinces/states that achieve adequate stool specimen collection§ from ≥80% of AFP cases.

Countries in the WHO AFR collect and review district-level data to make a qualitative determination of a country’s risk for an outbreak after a WPV importation or emergence of cVDPV and determine subnational areas at highest risk. A similar process is conducted by countries in the WHO EMR. The analysis described in this report is restricted to the 21 countries in the “WPV importation belt” in the WHO AFR based on 2012 data (Table 1). Countries were assessed to be at high risk for outbreaks during 2012–2013 based on proximity to countries where WPV was endemic or transmission was reestablished in 2012, current or recent civil unrest/insecurity, and any population immunity indicator in a high-risk tier. Countries were assessed to be at moderate risk based on any of the population immunity risk criteria suggesting vulnerability.

## Risk Mitigation Activities

Risk assessments have informed annual planning of activities to address the vulnerabilities of countries to improve population immunity through supplemental immunization activities (SIAs) and routine immunization, strengthen surveillance to promptly detect and investigate AFP cases, and enhance public health capacity to promptly respond after WPV importation.

### Immunization activities

To prevent transmission after WPV importation or cVPDV emergence, periodic preventive SIAs are undertaken nationally (national immunization days [NIDs]) and in portions of a country (subnational immunization days [SNIDs] in polio-free countries. In 2012, NIDs were conducted in seven of 10 high-risk countries and five of seven moderate-risk countries; SNIDs were conducted in eight of 10 high-risk countries and four of seven moderate-risk countries (Table 2). In 2013, NIDs have been conducted or are planned in eight of 10 high-risk countries and all seven moderate-risk countries; SNIDs have been conducted or are planned in seven of 10 high-risk countries and three of six moderate-risk countries (Table 2). The number of SIAs planned was influenced by proximity to Nigeria and Chad and assessed risk. The timing and scope of activities has been influenced by the availability of OPV preparations¶ and by the availability of funds in time to support them.

### Strengthening surveillance

Strengthening AFP surveillance to detect possible cases promptly after importation will lead to quicker programmatic response and limit the spread of WPV and cVDPVs. Periodic activities undertaken to strengthen AFP surveillance include national AFP surveillance reviews, program assessments, and surveillance training. During 2012–2013, national AFP surveillance reviews with technical staff from GPEI partners were conducted in eight of 10 high-risk countries and four of seven moderate-risk countries (Table 2). Training activities were undertaken or are planned in three high-risk or moderate-risk countries. In addition, external reviews in countries with prior outbreaks have been conducted at 3 and 6 months after outbreak confirmation and 6 months after the latest WPV or cVDPV case to assess the adequacy of the response and the ability of a program to detect continued transmission in a community. Country plans are developed as a result of the reviews, and follow-up visits are conducted to monitor the implementation of the recommendations.

### Editorial Note

Countries of the African “WPV importation belt” continue to be at risk for WPV outbreaks, as evident by the outbreak in the Horn of Africa that began April 2013. Assessment of the polio-free countries and subnational areas at higher risk for WPV transmission guides efforts to mitigate the impact of poliovirus transmission after WPV importations or emergence of VDPVs. The qualitative risk assessments allow the WHO Regional Office for Africa and other GPEI partners to prioritize SIA implementation. Risk assessments at the subnational level highlight underperforming areas to prioritize for targeted subnational SIAs. The value of preventive SIAs in risk mitigation in countries in the “WPV importation belt” is recently evident: the only importation-related outbreak identified globally in 2012 was in Niger, in which only a single WPV case was detected. Although risk assessments of countries of the WHO EMR had shown Somalia to be at high risk, security limitations prevented access to a large proportion of the population during the SIAs conducted before the outbreak was detected in May 2013. Additionally, in the 4 months since the outbreak was confirmed, preventive SIAs conducted in Kenya and Ethiopia, along with response SIAs conducted after the outbreak was detected, appear to have limited the WPV cases to certain high-risk border areas.

There are limitations associated with how risks are assessed, because many of the population immunity indicators are imprecise in indicating susceptibility overall and in particular, identifying pockets of underimmunized children; for this latter reason, many of the SIAs are not finely geographically targeted. Also, experience has indicated that SIA quality, assessed through the extent of planning, supervision, and delivery of poliovirus vaccine to a high proportion of children, tends to be lower in countries and areas where WPV has not recently circulated. Therefore, although SIAs enhance population immunity, they do not fully compensate for deficiencies in the delivery of health services and do not remove all risk.

SIA effectiveness has been improved by placing an increased emphasis on supervision and monitoring, which promotes greater accountability at the district and subdistrict levels (7). This has led to the identification and vaccination of children missed during previous SIAs and the formation of more detailed plans to improve subsequent SIAs. Better identification of repeatedly missed subpopulations, such as border populations and nomadic tribes, has led to more inclusive and detailed SIA plans, joint planning sessions between border districts, and improved synchronization of SIAs between countries (7,8).

Strengthening AFP surveillance will not decrease the risk for a WPV importation; however, prompt identification and rapid implementation of appropriate response efforts will limit the size of an outbreak (9). AFP surveillance performance indicators also have limitations in highlighting suboptimal surveillance (9). Although there are plans to extend environmental surveillance for polioviruses to some high-risk polio-free countries to improve the sensitivity of detecting poliovirus transmission and augment AFP surveillance, implementation of environmental surveillance requires substantial investment in personnel, supplies, and equipment to collect, process, and test specimens (9,10).

What is already known on this topic?

Nigeria remains the only polio-endemic country in Africa. However, multiple wild poliovirus (WPV) outbreaks have occurred in the World Health Organization (WHO) African Region (WHO AFR) since 2003, after the importation of indigenous West African WPV into 21 previously polio-free countries comprising a “WPV importation belt” that extends across the continent from the Sahara to the equator.

What is added by this report?

The Global Polio Eradication Initiative partners, including the WHO Regional Office for Africa, assess the risk for transmission of polioviruses after importation into polio-free countries and plan activities to decrease the risk for poliovirus transmission. Countries in the “WPV importation belt” in the WHO AFR deemed to be at high risk for outbreaks were primarily those located near countries with WPV cases in 2012 or with low population immunity indicators where routine vaccination coverage was suboptimal.

What are the implications for public health practice?

Suboptimal health infrastructure is a challenge in virtually all of the countries assessed as high-risk for poliovirus transmission. Insecurity and access barriers are additional challenges that will continue to threaten polio eradication efforts in the WHO AFR. All efforts to mitigate polio risks will need to continue as long as WPV transmission continues in polio-endemic countries. In addition, strengthening acute flaccid paralysis surveillance to promptly detect cases after importation will lead to quicker programmatic response and limit spread of WPV or circulating vaccine-derived polioviruses to other areas.

Mitigation activities are guided by periodic risk assessments. Plans are continually adapted based on the availability of funds, variation in the vaccine production cycle, and the changing epidemiology of WPV. These variables necessitate that GPEI partners make data-driven decisions to prioritize activities.

Throughout the WHO AFR, civil unrest and insecurity pose an increasing challenge for vaccination teams to access and reach children during SIAs. Insecurity has weakened routine immunization programs, hindered preventive SIAs, and limited AFP surveillance in large portions of countries, such as Mali and the Central African Republic during 2012–2013, and in many subnational areas throughout the region, including large parts of northern Nigeria. Partnering with relief organizations and implementing targeted SIAs as areas become accessible can assist in mitigating risks secondary to insecurity.

Going forward, GPEI partners are attempting to engage all development agencies in coordinated efforts to enhance childhood immunization services to optimize population immunity (10). Addressing funding limitations will be integral to ensuring that mitigation activities continue. All efforts to assess and mitigate risks will continue in the WHO AFR as long as endemic circulation of WPV is occurring in areas with low levels of population immunity.

## Figures and Tables

**FIGURE:**
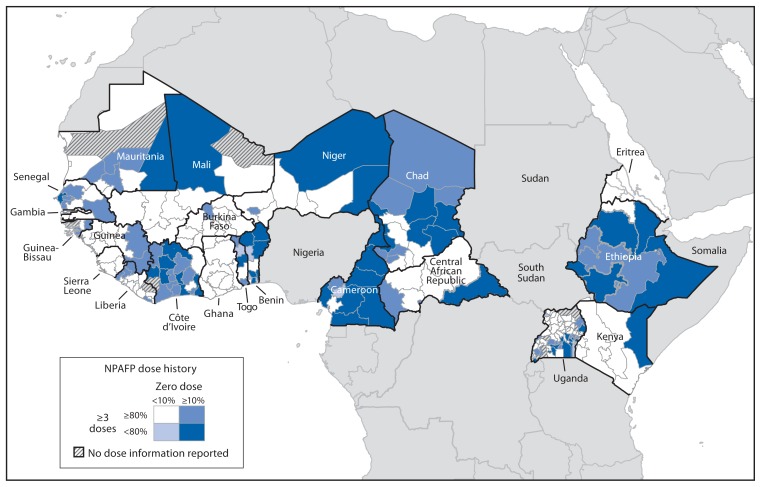
Oral poliovirus dose history among children aged 6–59 months with nonpolio acute flaccid paralysis (NPAFP) in countries of the “wild poliovirus importation belt” — World Health Organization African Region, 2012

**TABLE 1:** Key risk indicators for countries of the “wild poliovirus (WPV) importation belt,” by risk for transmission after an importation of WPV — World Health Organization (WHO) African Region, 2012–2013

	Population immunity indicators				Surveillance quality indicators		Other risk factors		
			
Country	National coverage with OPV3 (goal 90%)*	% districts that have ≥80% OPV3 coverage (goal 100%)†	Risk tier vaccine history ≥3 doses in children with NPAFP (Low, ≥90%; Moderate, 80%–90%; High, <80%)	Risk tier vaccine history zero doses in children with NPAFP (Low, <5%; Moderate, 5%–10%; High, >10%)	Risk tier for % provinces with NPAFP cases ≥2 per 100,000 (Low, >80%; Moderate, 51%–80%; High, ≤50%)§	Risk tier for % provinces with ≥80% of AFP cases with adequate specimens¶	Border a country with endemic or reestablished WPV transmission in 2012 (Nigeria, Chad)	No. of years with at least one importation event (2003–2012)	Areas of insecurity
**High-risk countries** **									
Benin	85%	95%	High	High	High	Low	Yes	4	No
Cameroon	85%	75%	High	High	High	Moderate	Yes	5	Yes
CAR	47%	13%	Moderate	Low	High	Low	Yes	5	Yes
Chad	56%	52%	High	High	High	Moderate	Yes	10	Yes
Côte d’ Ivoire	94%	NA	High	Moderate	High	Low	No	5	No
Ethiopia	70%	54%	High	Moderate	High	Moderate	No	4	Yes
Guinea-Bissau	78%	91%	High	Low	High	High	No	0	No
Mali	74%	73%	Low	Moderate	Low	Low	No	6	Yes
Niger	78%	98%	Low	Moderate	Moderate	Moderate	Yes	10	Yes
Uganda	82%	42%	High	Moderate	Moderate	Low	No	2	No
**Moderate-risk countries**									
Burkina Faso	90%	100%	Moderate	Moderate	High	Low	No	4	No
Guinea	57%	97%	Moderate	Low	Moderate	Low	No	3	No
Kenya	82%	NA	Low	Moderate	Moderate	Low	No	3	No
Liberia	77%	93%	Moderate	Low	Low	Low	No	2	No
Mauritania	80%	40%	Moderate	Moderate	Moderate	Low	No	2	Yes
Senegal	89%	NA	Moderate	Moderate	Moderate	Low	No	1	No
Togo	84%	89%	Moderate	Low	High	Low	No	3	No
**Low-risk countries**									
Eritrea	99%	48%	Low	Low	Low	Low	No	1	No
Gambia	98%	100%	Low	Low	Low	Low	No	0	No
Ghana	91%	80%	Low	Low	Low	Low	No	2	No
Sierra Leone	81%	100%	Low	Low	Low	Low	No	2	No

**Abbreviations:** OPV3 = ≥3 doses of oral poliovirus vaccine; NPAFP = nonpolio acute flaccid paralysis; AFP = acute flaccid paralysis; CAR = Central African Republic; NA = not available.

* WHO-United Nations Children’s Fund estimate (2012).

† Administrative data reported using a WHO-UNICEF Joint Reporting Form (2012).

§ The data for this indicator contain a substantial number of NPAFP cases with missing vaccination history. For 10 (46%) of 21 countries, ≥10% of the children with NPAFP had unknown vaccination histories.

¶ Standard WHO target is adequate stool specimen collection from ≥80% of AFP cases, in which two specimens are collected ≥24 hours apart, and within 14 days of paralysis onset, and arriving in good condition (received by reverse cold chain and without leakage or desiccation) in a WHO-accredited laboratory.

** Countries were assessed to be at high risk for outbreaks during 2012–2013 based on proximity to countries with WPV-endemic or reestablished transmission in 2012, current or recent civil unrest, or with any population immunity indicator in a high-risk tier. Countries were assessed to be at moderate risk based on any of the population immunity risk criteria suggesting vulnerability.

**TABLE 2:** Risk mitigation activities in countries of the “wild poliovirus (WPV) importation belt” — World Health Organization African Region, 2012–2013

	Supplementary immunization activities: national and subnational immunization days (NID/SNID)			Surveillance strengthening activities: national surveillance reviews (SR) program assessments (PA)* or surveillance training activities (ST)	
		
Importation countries	Jan 2012–Dec 2012 (NID/SNID)	Jan 2013–June 2013 (NID/SNID)	July 2013–Dec 2013 (NID/SNID) (planned)	Jan 2012–June 2013	July 2013–Dec 2013 (planned)
**High-risk countries** †					
Benin	3/0	2/0	2/0	SR	
Cameroon	0/3	0/2	1/2	SR	PA, ST
Central African Republic	5/2	0/0	1/2	SR	PA, ST
Chad	3/11	3/2	2/4	SR, ST	PA
Côte d’ Ivoire	4/0	2/0	1/0	SR	
Ethiopia	0/3	0/2	2/3		
Guinea-Bissau	1/0	1/0	1/0	SR	
Mali	1/6	2/1	2/1	SR	
Niger	4/5	2/2	2/2	SR, PA	ST
Uganda	0/1	0/0	0/2		
**Moderate-risk countries**					
Burkina Faso	4/1	2/1	2/0	SR	
Guinea	4/1	2/0	1/0	SR	
Kenya	0/6	0/2	2/6		PA, ST
Liberia	3/0	2/0	1/0	PA	
Mauritania	3/0	1/0	1/0	SR	
Senegal	1/0	1/0	1/0	SR	
Togo	1/0	1/0	1/0		
**Subtotal (risk countries)**	**37/39**	**21/12**	**23/22**		
**Low-risk countries**					
Eritrea	1/0	0/0	2/0		
Gambia	1/0	1/0	1/0		
Ghana	1/0	1/0	1/0	SR	
Sierra Leone	3/0	0/0	1/0		
**Total**	**6/0**	**2/0**	**5/0**		

* Program assessments include assessments done after the occurrence of a case of WPV or circulating vaccine-derived poliovirus and planned comprehensive immunization program reviews.

† Countries were assessed to be at high risk for outbreaks during 2012–2013 based on proximity to countries with WPV-endemic or reestablished transmission in 2012, current or recent civil unrest, or with any population immunity indicator in a high-risk tier. Countries were assessed to be at moderate risk based on any of the population immunity risk criteria suggesting vulnerability.

## Footnotes

* The 21 “WPV importation belt” countries in the WHO AFR with either WPV outbreaks of West African origin since 2003 or with two or more contiguous borders with countries with WPV outbreaks are Benin, Burkina Faso, Cameroon, Central African Republic, Chad, Côte d’Ivoire, Eritrea, Ethiopia, Gambia, Ghana, Guinea, Guinea-Bissau, Kenya, Liberia, Mali, Mauritania, Niger, Senegal, Sierra Leone, Togo, and Uganda. Gambia and Guinea-Bissau have not reported WPV cases since before 2000.

† Somalia is in the WHO EMR. Other EMR countries on the African continent that have had outbreaks attributable to WPV importations of West African origin since 2003 are South Sudan, which will join the WHO AFR in 2014, and Sudan.

§ Two specimens collected ≥24 hours apart within 14 days of paralysis onset, and arriving in good condition (received using reverse cold chain and without leakage or desiccation) in a WHO-accredited laboratory.

¶ All OPV is obtained from manufacturers that have been “prequalified” by WHO for purchase by the United Nations Children’s Fund. Because of the exit of one manufacturer before another could begin larger-scale production, a shortage of OPV preparations occurred in 2012. tOPV is the preparation of choice in routine childhood vaccination in countries that have not introduced inactivated poliovirus vaccine. Monovalent vaccines (types 1 or 3) were predominantly used in SIAs during 2005–2011 to selectively increase population immunity against a given type. With the introduction of bivalent OPV (bOPV) types 1 and 3 in 2012 after the demonstration of noninferiority to monovalent vaccines, bOPV is now predominantly used in SIAs, and manufacturers give its production priority.
